# Post-COVID-19 pulmonary fibrosis and its predictive factors: a prospective study

**DOI:** 10.1186/s43055-021-00632-9

**Published:** 2021-10-05

**Authors:** Mehrdad Nabahati, Soheil Ebrahimpour, Reza Khaleghnejad Tabari, Rahele Mehraeen

**Affiliations:** 1 Department of Radiology, Shahid Beheshti Hospital, Babol University of Medical Sciences, Ganjafrooz Street, Babol, Mazandaran 47176-47745 Iran; 2 Infectious Diseases and Tropical Medicine Research Center, Health Research Institute, Babol University of Medical Sciences, Babol, Iran; 3 Department of Radiology and Radiotherapy, School of Medicine, Babol University of Medical Sciences, Babol, Iran

**Keywords:** Computed tomography, Coronavirus Disease 2019, SARS-CoV-2, Pulmonary fibrosis

## Abstract

**Background:**

We aimed to prospectively assess the lung fibrotic-like changes, as well as to explore their predictive factors, in the patients who survived Coronavirus Disease 2019 (COVID-19) infection. In this prospective cross-sectional study, we recruited patients who had been treated for moderate or severe COVID-19 pneumonia as inpatients and discharged from Rohani hospital in Babol, northern Iran, during March 2020. The clinical severity of COVID-19 pneumonia was classified as per the definition by World Health Organization. We also calculated the CT severity score (CSS) for all patients at admission. Within the 3 months of follow-up, the next chest CT scan was performed. As the secondary outcome, the patients with fibrotic abnormalities in their second CT scan were followed up in the next 3 months.

**Results:**

Totally, 173 COVID-19 patients were finally included in the study, of whom 57 (32.9%) were male and others were female. The mean age was 53.62 ± 13.67 years old. At 3-month CT follow-up, evidence of pulmonary fibrosis was observed in 90 patients (52.0%). Consolidation (odds ratio [OR] = 2.84), severe disease (OR 2.40), and a higher CSS (OR 1.10) at admission were associated with increased risk of fibrotic abnormalities found at 3-month CT follow-up. Of 62 patients who underwent chest CT scan again at 6 months of follow-up, 41 patients (66.1%) showed no considerable changes in the fibrotic findings, while the rest of 21 patients (33.9%) showed relatively diminished lung fibrosis.

**Conclusion:**

Post-COVID-19 lung fibrosis was observed in about half of the survivors. Also, patients with severe COVID-19 pneumonia were at a higher risk of pulmonary fibrosis. Moreover, consolidation, as well as a higher CSS, in the initial chest CT scan, was associated with increased risk of post-COVID-19 lung fibrosis. In addition, some patients experienced diminished fibrotic abnormalities in their chest CT on 6-month follow-up, while some others did not.

## Background

Coronavirus Disease 2019 (COVID-19), which is caused by severe acute respiratory syndrome coronavirus 2 (SARS-CoV-2), quickly spread across the world since its outbreak in China began in December 2019 [1]. By June 25, 2021, about 180 million cases have been confirmed with this disease worldwide, including about 4 million deaths [2]. Patients with COVID-19 infection have different symptoms, specifically respiratory symptoms that can manifest as severe pneumonia and acute respiratory distress syndrome [3]. Moreover, it has been reported that some cases who recovered from the disease with negative laboratory tests still suffer from persistent symptoms (from mild to severe forms of respiratory distress requiring long-term oxygen therapy) due to residual sequelae (e.g., pulmonary fibrosis) [4, 5].

Chest computed tomography (CT) scan has an important role in the diagnosis and follow-up of the COVID-19 patients. Various CT findings have been reported related to COVID-19 pneumonia with mild to severe lung involvement. Some previous studies also alluded to fibrotic consequences following the infection, which is a considerable issue for the patients’ clinical outcomes [5, 6]. For example, a study reported that about one third of the COVID-19 survivors showed fibrotic abnormalities in their CT scans within the 6-month follow-up [7].

Identifying the predictive factors for the post-COVID-19 lung fibrosis can possibly help in management of such a serious complication through controlling the risk factors and/or administrating the anti-fibrotic drugs in high-risk cases. In the present study, we aimed to prospectively assess the lung fibrotic-like changes, as well as to explore their predictive factors, in the patients who survived COVID-19 infection.

## Methods

### Locations and patients

In this prospective cross-sectional study, we recruited patients who had been treated for moderate or severe COVID-19 pneumonia as inpatients and discharged from Rohani hospital in Babol, northern Iran, during March 2020. The SARS-CoV-2 infection was confirmed by real-time polymerase chain reaction (RT-PCR) on nasopharyngeal swab samples collected from cases initially presented with suspicious symptoms (e.g., fever, cough, dyspnea, sputum discharge, etc.). These patients also underwent chest CT scan at admission. The clinical severity of COVID-19 pneumonia was classified as moderate (evidence of lower respiratory disease with oxygen saturation ≥ 94%) and severe (oxygen saturation < 94%, a ratio of arterial partial pressure of oxygen to fraction of inspired oxygen < 300, respiratory rate > 30, or lung infiltrates > 50%) as per the definition by World Health Organization (WHO) [8]. Patients with mild disease were not admitted as per the national COVID-19 protocol.

After assessing the patients’ medical records, the required data were extracted, including demographic information (such as sex and age), symptoms, comorbidities (such as cardiovascular diseases [CVDs], asthma, chronic obstructive pulmonary disease [COPD], and diabetes). The criteria for discharging were based on the flowchart of diagnosis and treatment of COVID-19 disease in Iran (improved general conditions, increased oxygen saturation without respiratory distress, suppressed fever for at least three days, improved laboratory results) [9]. Within the 3 months of follow-up (in June 2020), the next chest CT scan was performed on the patients who had residual symptoms and/or would like to monitor the changes of their CT images. The exclusion criteria were incomplete information about RT-PCR results or comorbidities, as well as unwillingness to participate in the study. We also did not include patients with history of smoking to prevent its potential confounding effects on the study outcomes. As the secondary outcome, the patients with fibrotic abnormalities in their second CT scan were followed up in the next 3 months (in September 2020) to monitor their imaging changes.

### Imaging procedures

Non-enhanced 16-detector-row CT scans were conducted on the patients in the supine position during deep inspiration breath-hold from the thoracic inlet to the diaphragm (siemens SOMATOM Emotion 16, Siemens Healthcare, Med Image Systems, Germany). The following scanning parameters were used: tube voltage, 100 kV for patients with BMI ≤ 30 and 120 kV for patients with BMI > 30; tube current, 50–100 mAs; pitch, 0.8–1.5; thickness, 1–3 mm; Matrix, 512. No additional image reconstructions were necessary. The CT scans (at both admission and follow-up) were conducted using the same scanners and assessed by two senior radiologists with experience of more than 15 years (R.M. and M.N.), who was not aware of the patients’ status. CT imaging features, including traction bronchiectasis, honeycombing, parenchymal bands, and interlobar septal thickening (IST), were considered as the fibrotic-like changes. Also, parenchymal bands and interlobar septal thickening were considered as mild/moderate fibrosis, and traction bronchiectasis and honeycombing were considered as severe fibrosis.

We also calculated the CT severity score (CSS) for all patients at admission, on the basis of the involvement of each five lung zones, which was as follows [10]: score 0, no involvement; score 1, < 5% involvement; score 2, 5–25% involvement; score 3, 26–50% involvement; score 4, 51–75% involvement; and score 5, > 75% involvement. Finally, the total CSS was calculated by summing the scores, ranging from 0 to 25.

### Data analysis

We used descriptive analysis to calculate frequencies, percentages, mean, and standard deviations. Kolmogorov–Smirnov test was used to evaluate the normality of the data. Independent t-test and Mann–Whitney test were used for comparing parametric and nonparametric continuous data between the groups, respectively. We conducted chi-squared test and logistic regression analysis to investigate the association of baseline information and imaging findings of the patients with post-COVID-19 lung fibrosis. The factors with significant association (consolidation and severe disease) were entered into the multivariable analysis. Concerning CSS, we presented it as both of continuous variable and median with interquartile range (IQR). The results were presented as odds ratio (OR) as well as 95% confidence interval (CI). We also estimated the area under ROC curve (AUC) for predictive ability of the CT scan features. A *p* value less than 0.05 was considered statistically significant. All statistical analyses were performed by SPSS software.

## Results

### Patients’ information at admission

A total of 173 COVID-19 patients were finally included in the study for further investigations, of whom 57 (32.9%) were male and 116 (67.1%) were female. The mean age was 53.62 ± 13.67, ranging from 18 to 93 years old. The symptoms of the patients included fever (78.6%), chills (67.6%), dry cough (63.0%), dyspnea (57.2%), myalgia (56.1%), sputum (37.0%), sore throat (27.2%), and headache (18.5%). Cardiovascular diseases were the most prevalent comorbidity seen in the cases (*n* = 72, 41.6%). In the initial chest CT scan, ground glass opacity was seen in 136 patients (78.6%), consolidation in 141 patients (81.5%), and crazy paving in 31 patients (17.9%). Distribution of basic information and initial CT findings between the two groups with and without fibrosis are summarized in Table 1.

**Table 1:** Comparison of basic information and initial CT findings between the two groups

Variables	With lung fibrosis (*n* = 90)	Without lung fibrosis (*n* = 83)	*p* value
Age (years), mean ± SD	54.66 ± 13.05	52.51 ± 14.30	0.303
Male gender, *n* (%)	34 (37.8)	23 (27.7)	0.159
Comorbidity, *n* (%)			
CVDs	35 (38.9)	37 (44.6)	0.448
Asthma/COPD	11 (12.2)	4 (4.8)	0.083
Diabetes	12 (13.3)	15 (18.1)	0.391
Severe disease^*^, *n* (%)	59 (65.6)	34 (41.0)	0.001
Imaging findings, *n* (%)			
Ground glass	68 (75.6)	68 (81.9)	0.307
Consolidation	81 (90.0)	60 (72.3)	0.003
Crazy paving	17 (18.9)	14 (16.9)	0.729
CSS (continuous), median (IQR)	21 (15–23)	18 (14–21)	0.002
CSS ≥ 19, *n* (%)	57 (63.3)	37 (44.6)	0.014

CVDs, cardiovascular diseases; COPD, chronic obstructive pulmonary disease; CSS, CT severity score; IQR, interquartile range

*Based on the WHO definition

### Three-month follow-up findings

On follow-up CT scans, evidence of pulmonary fibrosis was observed in 90 patients (52.0%), including parenchymal bands (in 58 patients, 33.5%), IST (in 75 patients, 43.4%), bronchiectasis (in 11 patients, 6.4%), and honeycombing (in 4 patients, 2.3%). No differences were found between the groups with and without fibrosis in terms of age, gender, and comorbidity.

Table 2 shows the factors associated with post-COVID-19 lung fibrosis according to the logistic regression analysis. As represented, severe disease (classified as per the WHO definition) was associated with increased risk of pulmonary fibrosis at follow-up (OR 2.74, 95% CI 1.48–5.08). Also, patients who had consolidation in their initial CT scan were at a higher risk of post-COVID-19 lung fibrosis (OR 3.45, 95% CI 1.49–7.99). Moreover, patients with pulmonary fibrosis had a higher CSS than those without (OR 1.10, 95% CI 1.03–1.18). The median number of CSS was 19 (interquartile range: 14–22), and we used it as a threshold for the relevant analyses. Based on the analyses, CSS ≥ 19 could predict post-COVID-19 lung fibrosis in the study cases (OR 2.15). In multivariable analysis, consolidation (OR 2.84, 95% CI 1.20–6.73, *p* = 0.018) and severe disease (OR 2.40, 95% CI 1.27–4.51, *p* = 0.007) were still associated with increased risk of fibrotic abnormalities, with AUC = 58.9% and AUC = 62.3%, respectively.

**Table 2:** Factors associated with post-COVID-19 lung fibrosis in logistic regression model

Variables	Odds ratio (95% confidence interval)	*p* value
Disease severity*		
Moderate	1	
Severe	2.74 (1.48–5.08)	0.001
Consolidation		
No	1	
Yes	3.45 (1.49–7.99)	0.003
CT severity score (continuous)	1.10 (1.03–1.18)	0.008
CT severity score ≥ 19	2.15 (1.17–3.95)	0.014

*Based on the WHO definition

**Fig. 1:**
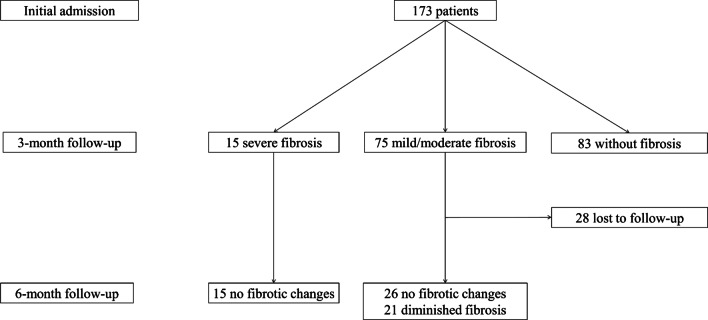
Lung fibrotic-like changes in the COVID-19 patients at different follow-ups

**Fig. 2:**
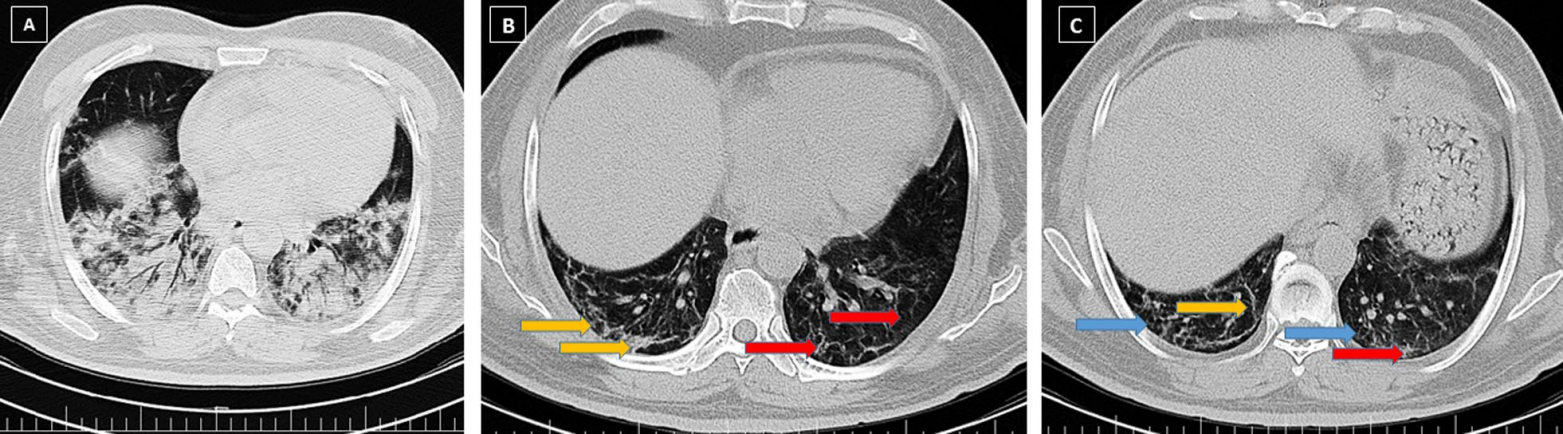
A 55-year-old male patient presented with fever, dyspnea and sputum discharge, who was admitted to hospital and was discharged on day 19 of admission. Ground glass opacities and consolidations in multiple pulmonary lobes are observed in the initial CT scan (**A**). The total CT severity score was calculated as 23, and the disease severity was severe. Three-month (**B**) and 6-month (**C**) follow-up CT shows parenchymal bands (yellow arrows), interlobular septal thickening (red arrows), and vacuolation (blue arrows), which were persistent findings without any significant changes after 3 months

**Fig. 3:**
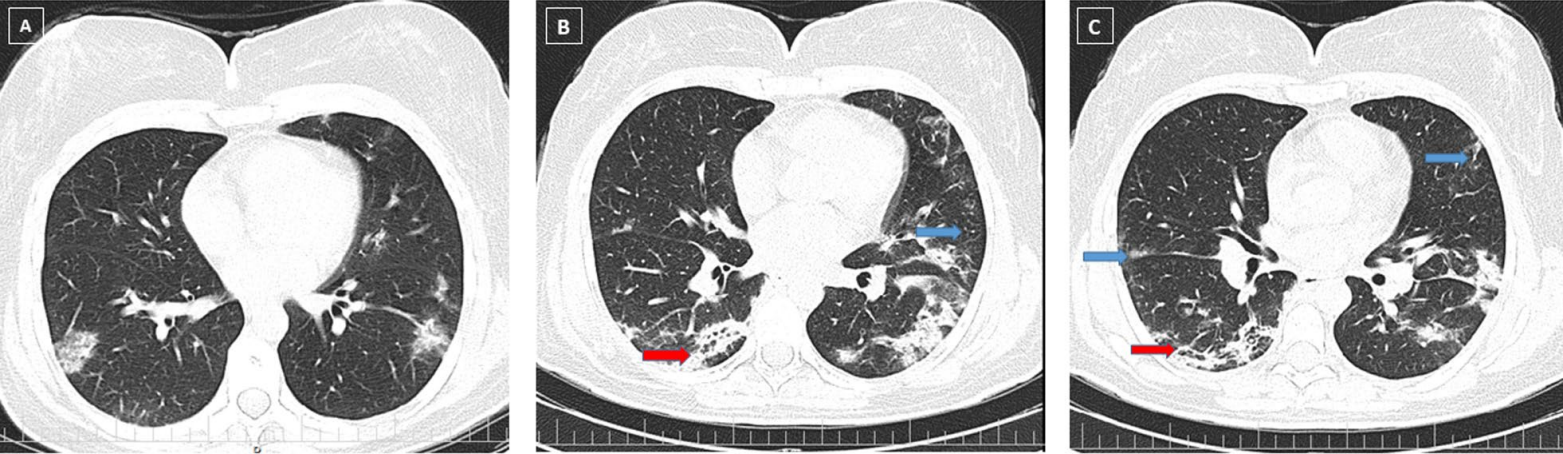
A 46-year-old female patient presented with fever and dyspnea, who was admitted to hospital and was discharged on day 15 of admission. Ground glass opacities and consolidations in several pulmonary lobes are observed in the initial CT scan (**A**). The total CT severity score was calculated as 14, and the disease severity was moderate. Three-month follow-up chest CT (**B**) shows some parenchymal bands and vacuolation (red arrow) and interlobular septal thickening (blue arrow) in several lobes. Six-month follow-up chest CT (**C**) shows a few parenchymal bands (red arrow) and interlobular septal thickening (blue arrow), which was diminished in comparison to the previous CT

**Fig. 4:**
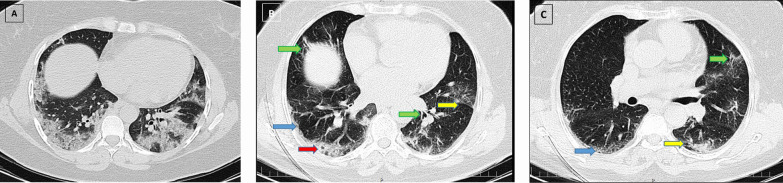
A 47-year-old female patient presented with fever and cough, who was admitted to hospital and was discharged on day 16 of admission. Ground glass opacities and consolidations in multiple pulmonary lobes are observed in the initial CT scan (**A**). The total CT severity score was calculated as 18, and the disease severity was moderate. Three-month follow-up chest CT (**B**) shows a parenchymal band (yellow arrow), interlobular septal thickening (blue arrow), mild honeycombing (red arrow), and bronchiectasis (green arrows). Six-month follow-up chest CT (**C**) shows a few parenchymal bands (yellow arrow) and interlobular septal thickening (blue arrow) and mild bronchiectasis (green arrow), which was diminished in comparison to the previous CT

**Fig. 5:**
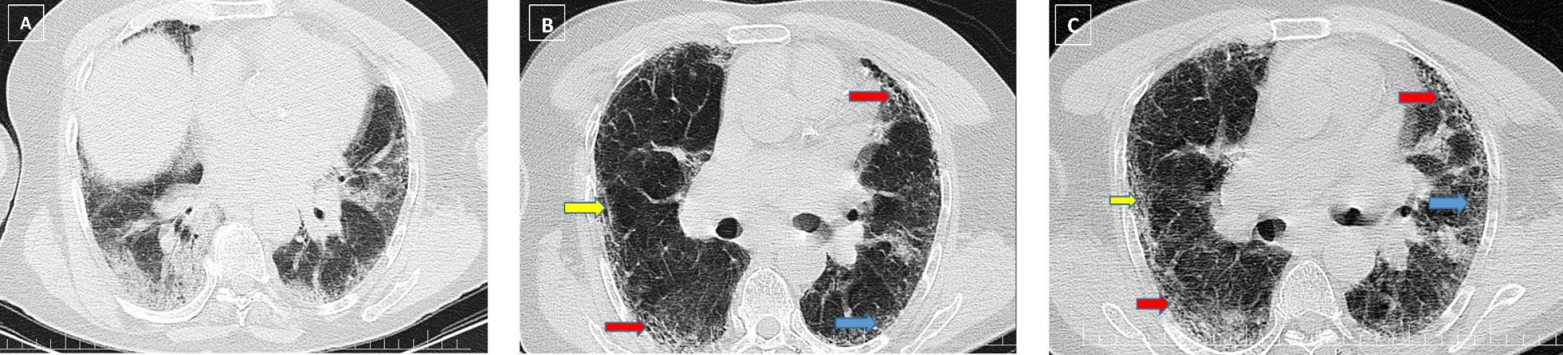
A 70-year-old male patient presented with fever, chills, cough, and myalgia, who was admitted to hospital and was discharged on day 18 of admission. Ground glass opacities and consolidations in multiple pulmonary lobes are observed in the initial CT scan (**A**). The total CT severity score was calculated as 21, and the disease severity was moderate. Three-month follow-up chest CT (**B**) shows parenchymal bands (yellow arrow) and interlobular septal thickening (blue arrow) and honeycombing (red arrow). Six-month follow-up chest CT (**C**) shows parenchymal bands (yellow arrow) and interlobular septal thickening (blue arrow) and honeycombing (red arrow) as well

After categorizing fibrosis severity into mild/moderate and severe, it was observed that 13.6% (*n* = 15) of fibrotic patients had severe fibrosis (Fig. 1). Also, no significant differences were found between the two groups of fibrosis severity in terms of study variables, such as demographic, clinical, and imaging information. In Figs. 2, 3, 4, and 5, the 3-month CT follow-ups were represented.

### Six-month follow-up findings

Out of 90 patients with evidence of pulmonary fibrosis in the first 3 months of follow-up, 62 patients (68.2%) underwent chest CT scan again at 6 months of follow-up in order to recheck the previous CT findings (Table 3). It was found that fibrotic findings were not considerably changed in 41 patients (66.1%), of whom 15 patients had severe lung fibrosis. On the other hand, lung fibrosis was relatively diminished in 21 patients (33.9%), who all had mild/moderate lung fibrosis. No progressive signs were seen for the fibrotic abnormalities in any patients (Fig. 1). In Figs. 2, 3, 4 and 5, the 6-month CT follow-ups were represented.

**Table 3:** Lung fibrotic-like changes in chest CT scan at three- and six-month follow-ups

Imaging findings	Three-month follow-up (*n* = 90)	Six-month follow-up (*n* = 62)
Parenchymal bands, *n* (%)		
No	32 (35.6)	40 (64.5)
Yes	58 (64.4)	22 (35.5)
Interlobular septal thickening, *n* (%)		
No	15 (16.7)	36 (58.1)
Yes	75 (83.3)	26 (41.9)
Bronchiectasis, *n* (%)		
No	79 (87.8)	51 (82.3)
Yes	11 (12.2)	11 (17.7)
Honeycombing, *n* (%)		
No	86 (95.6)	58 (93.5)
Yes	4 (4.4)	4 (6.5)

## Discussion

In the present study, we investigated the development of lung fibrotic-like changes in 90 patients who recovered from the moderate or severe COVID-19 pneumonia within three- and 6-month follow-up. As stated, about half of the cases showed an evidence of fibrotic abnormalities on the 3 months follow-up, of whom 13.6% had severe fibrosis. We also found that lung fibrosis was not considerably changed in 41 patients of 62 patients who underwent chest CT scan again at 6 months of follow-up, while was relatively diminished in 21 patients. To the best of our knowledge, the present survey was the first report of post-COVID-19 lung fibrosis in our region. So far, various studies alluded to the manifestations of COVID-19 during follow-up; However, limited number of studies focused on the lung fibrosis as the main outcome, which can lead to permanent adverse outcomes in the survivors, such as irreversible pulmonary dysfunction [11].

In the study by Han et al. [7], fibrotic abnormalities were seen in 35% of the patients over the 6 months of follow-up, which was lower than the results obtained by us. Also, other study by Ali et al. [12] showed a rate of 32% for pulmonary fibrosis in the COVID-19 patients within 3-month follow-up, which was less than that we found in this study. These variations could be explained by differences in studies population and paraclinical measures and management by technicians and clinicians.

We also found that patients who had consolidation, as well as a higher CSS, in their initial chest CT scan, were at a higher risk of post-COVID-19 pulmonary fibrosis compared with those without. Furthermore, it was demonstrated that severe COVID-19 pneumonia increased risk of fibrotic lung damages in the patients. In the study by Ali et al. [12], it was declared that older age, cigarette smoking, higher CSS, and long-term mechanical ventilation were associated with increased risk of lung fibrosis. The same results for age, CSS, and mechanical ventilation were seen in the study by Han et al. [7] as well. Therefore, identifying and controlling these predictors in clinical practice can help in preventing the development of and/or reducing the progression of the lung fibrosis as a considerable adverse outcome of COVID-19 pneumonia.

The main cause of post-COVID-19 pulmonary fibrosis still remains unclear; However, some theories allude to the abnormal immune mechanisms and the resultant cytokine storm [13]. Also, more studies need to be done to clarify why some patients develop lung fibrosis, while some others not.

It should be stated that there is not a consensus on the use of anti-fibrotic drugs in the prevention and treatment of lung fibrosis in the COVID-19 survivors yet. These drugs can decrease pulmonary damage in the high-risk patients and are presently used for interstitial lung diseases [13]. Considering that lung fibrosis is accepting as an important adverse outcome in the survivors of COVID-19, it is suggested to reach a consensus on putting anti-fibrotic drugs into the COVID-19 treatment guidelines, specifically concerning the high-risk patients.

Although we tried to assess the status of lung fibrosis in the two follow-up times (3 and 6 months), further studies should assess the patients in longer follow-ups to find out if the fibrotic abnormalities are temporary or permanent. Also, studies with larger sample size are proposed to be carried out in the future.

## Conclusion

According to the results, post-COVID-19 lung fibrosis was observed in about half of the survivors. Also, patients with severe COVID-19 pneumonia were at a higher risk of pulmonary fibrosis. Moreover, consolidation, as well as a higher CSS, in the initial chest CT scan, was associated with increased risk of post-COVID-19 lung fibrosis. In addition, some patients experienced diminished fibrotic abnormalities in their chest CT on 6-month follow-up, while some others did not. Identifying and controlling these predictive factors, as well as evaluating the therapeutic position of the anti-fibrotic drugs, in clinical practice can help in preventing the development of and/or reducing the progression of the lung fibrosis as a considerable adverse outcome of COVID-19 pneumonia.

## Data Availability

The datasets during and/or analyzed during the current study are available from the corresponding author on a reasonable request.

## Abbreviations

COVID-19: Coronavirus Disease 2019
SARS-CoV-2: Severe acute respiratory syndrome coronavirus 2
RT-PCR: Real-time polymerase chain reaction
CT: Computed tomography
CVDs: Cardiovascular diseases
COPD: Chronic obstructive pulmonary disease
WHO: World Health Organization
CSS: CT severity score
IST: Interlobar septal thickening
OR: Odds ratio
AUC: Area under the curve
ROC: Receiver operating characteristic
